# Clinical Lectures on Diseases of the Ear

**Published:** 1873-01

**Authors:** E. L. Holmes

**Affiliations:** Chicago


A MONTHLY RECORD OF

*Medicine, Surgery and the Collateral Sciences.*

EDITED BY J. ADAMS ALLEN, M.D., LL.D.; AND WALTER HAY, M.D.

VOL. XXX. — JANUARY, 1873. — NO. 1.

### Original Communications.

ARTICLE I.—*Clinical Lectures on Diseases of the Ear.* By E. L. HOLMES, M.D., Chicago.

### THE MIDDLE EAR AND ITS DISEASES.

*Gentlemen :*

It will not be unprofitable to review briefly the remarks I have made from time to time, as patients with diseases of the tympanum have come to the clinic for treatment; for these diseases are very common, and should engage your serious attention.

You have studied the principal affections of the external meatus. Diseases of the labyrinth, which are relatively quite rare, and unfortunately seldom curable, have also fallen under your observation.

What you have already investigated at this clinic, is sufficient, I think, to correct the opinion which you may have had, and which I think is generally held by practitioners, that the classification of aural diseases is very arbitrary and unsatisfactory. You must now admit, I trust, that the classification is strictly founded on an anatomical, physiological basis, and easily comprehended by any one acquainted with the anatomy of the ear.

The middle ear, or tympanum, is an extremely irregular cavity—a continuation, as it were, of the external meatus, surrounded on almost every side by bone. Its average length is about six and a half lines; its height varies in different portions from three and a half to seven and a half lines. The distance from the membrana tympani to the bony wall opposite to it, varies from one to two lines.

The external lateral wall is the membrana tympani—the internal lateral wall is the bony covering of the labyrinth. The roof of this cavity, over which lies the brain, is a thin septum of bone, through which are several minute openings for the passage of vessels.

The floor of the tympanum is also in some portions a thin septum separating it from the jugular vein. This septum, as also the roof, is sometimes in places destitute of bone, as if ossification had been incomplete.

The posterior wall separates the tympanum from the mastoid cells. In the upper part of this wall is the passage to these cells.

The upper portion of the anterior wall contains the mouth of the Eustachian tube.

The inner lateral wall is an important portion of the tympanum. In it are situated posteriorly the foramina ovale and rotundum—the former opening into the vestibule, the latter opening into the cochlea. Anterior to these, and nearly opposite the centre of the membrana tympani, is the promontory—an elevation produced by one of the coils of the cochlea. In front of this promontory is the portion of the wall covering the carotid artery.

There are two exceedingly minute muscles in the middle ear—one attached to the stapes, and called the stapedius; the other attached to the handle of the malleus, called the tensor membranæ tympani.

These muscles are believed to control, to a certain extent, the tension of the membrana tympani, and to modify the degree with which the stapes presses upon the fluid of the labyrinth.

Never forget the character of the tissues surrounding this small cavity. Calling to mind the patients who have come here with long-continued purulent discharge from the ears, and with destruction of the membrana tympani, you must have often asked yourselves how such severe disease, so very near vital organs, could continue so long and violent without fatal results. It is well known that purulent discharge from the ear may result in death. For this reason you should never permit a case with obscure cerebral symptoms to be under your care without a careful examination of the ears. This should be impressed on your minds the more earnestly, because you will be in danger of overlooking it; for in proportion to the number of cases of otorrhœa, we must confess that fatal cases are quite rare.

To comprehend why diseases of the middle ear so often impair hearing, you need but recur to the delicate mechanism of the minute chain of bones extending across the middle ear, and of the membrane of the foramen rotundum.

Remember that these structures, so wonderfully connected, are made to vibrate by the waves of sound—that by them the fluid in the labyrinth is set in motion, which is communicated to the microscopic fibres of the auditory nerve, producing the sensation we call sound.

In the ear, as in all other organs, inflammation is the most frequent source of injury. If the products of inflammation, mucus or pus, collect in the middle ear; if the mucous membrane covering the membrana tympani, the membrane of the foramen rotundum or the ossicles, becomes œdematous, or indurated, as the result of inflammation, this apparatus can no longer vibrate so sensitively as in the normal condition.

Even when the quantity of air in the middle ear is diminished by obstruction of the Eustachian tube, the vibratory motion of the membrana tympani is impeded by the external atmosphere crowding unduly upon it. Hence, as you are aware, the manner in which disease of the tympanum, in its various forms, impairs hearing, is almost invariably by impairing motion of the mechanism above described.

The simplest classification of the diseases of the middle ear is perhaps the following: Acute—chronic catarrh; Acute—chronic purulent otitis; which we will consider at another time.

